# Internet-guided cognitive, behavioral and chronobiological interventions in depression-prone insomnia subtypes: protocol of a randomized controlled prevention trial

**DOI:** 10.1186/s12888-020-02554-8

**Published:** 2020-04-15

**Authors:** Jeanne Leerssen, Jessica C. Foster-Dingley, Oti Lakbila-Kamal, Laura M. S. Dekkers, Anne C. W. Albers, Savannah L. C. Ikelaar, Teodora Maksimovic, Rick Wassing, Simon J. Houtman, Tom Bresser, Tessa F. Blanken, Bart te Lindert, Jennifer R. Ramautar, Eus J. W. Van Someren

**Affiliations:** 1 Department of Sleep and Cognition, Netherlands Institute for Neuroscience (NIN), an institute of the Royal Netherlands Academy of Arts and Sciences, Meibergdreef 47, 1105 BA Amsterdam, The Netherlands; 2 Department of Integrative Neurophysiology, Center for Neurogenomics and Cognitive Research (CNCR), Amsterdam Neuroscience, VU University Amsterdam, Amsterdam, The Netherlands; 3 Amsterdam UMC, Vrije Universiteit, Psychiatry, Amsterdam Neuroscience, Amsterdam, The Netherlands

**Keywords:** Insomnia, Depression, Cognitive behavioral therapy for insomnia, Chronobiological therapy, Randomized controlled trial

## Abstract

**Background:**

Major depressive disorder is among the most burdening and costly chronic health hazards. Since its prognosis is poor and treatment effectiveness is moderate at best, prevention would be the strategy of first choice. Insomnia may be the best modifiable risk factor. Insomnia is highly prevalent (4–10%) and meta-analysis estimates ±13% of people with insomnia to develop depression within a year. Among people with insomnia, recent work identified three subtypes with a particularly high lifetime risk of depression. The current randomized controlled trial (RCT) evaluates the effects of internet-guided Cognitive Behavioral Therapy for Insomnia (CBT-I), Chronobiological Therapy (CT), and their combination on insomnia and the development of depressive symptoms.

**Methods:**

We aim to include 120 participants with Insomnia Disorder (ID) of one of the three subtypes that are more prone to develop depression. In a two by two factorial repeated measures design, participants will be randomized to CBT-I, CT, CBT-I + CT or treatment as usual, and followed up for one year. The primary outcome is the change, relative to baseline, of the severity of depressive symptoms integrated over four follow-ups spanning one year. Secondary outcome measures include a diagnosis of major depressive disorder, insomnia severity, sleep diaries, actigraphy, cost-effectiveness, and brain structure and function.

**Discussion:**

Pre-selection of three high-risk insomnia subtypes allows for a sensitive assessment of the possibility to prevent the development and worsening of depressive symptoms through interventions targeting insomnia.

**Trial registration:**

Netherlands Trial Register (NL7359). Registered on 19 October 2018.

## Background

Major depressive disorder is among the most burdening and costly chronic health hazards. Its prognosis is poor and treatment effectiveness is at best moderate [1]. The Global Consortium for Depression Prevention concluded that our best chance to combat the global burden of depression is to provide preventive intervention to people at risk [2]. Insomnia plays an important role in developing and maintaining depressive symptoms [3] and may be the best modifiable risk factor of depression [4–7]. It has recently been revealed that insomnia comes in five different stable subtypes, of which three have an increased risk of lifetime depression [8]. The current randomized controlled trial (RCT) therefore evaluates the effects of internet-guided Cognitive Behavioral Therapy for Insomnia (CBT-I), Chronobiological Therapy (CT), and their combination on insomnia and the development of depressive symptoms in people with the type of Insomnia Disorder (ID) that are most prone to develop depression.

With a prevalence of 4–10% in the general population, ID is the second most prevalent mental disorder [9]. ID increases the use of health care facilities and contributes to cognitive and health problems including the risk of developing obesity, diabetes, cardiovascular disease and most markedly, of depression [10]. Meta-analysis estimates that 13% of people with ID will develop depression within a year [5]. Not all people with ID are equally likely to develop depression. It has recently been discovered that ID is not a uniform disorder. Rather, people with ID represent a heterogeneous mix of at least five different subtypes, of which three are more prone to developing depression [8]. It is unknown which environmental, psychological, or biological markers of vulnerability underlie the subtypes of ID that are prone to develop depression. Identifying these markers could significantly contribute to our understanding of why some people suffering from ID are vulnerable to developing a depression, while others are resilient.

The most common approach for the treatment of ID is pharmacological therapy [11]. However, pharmacological treatments have a high prevalence of negative side effects, such as daytime drowsiness, risk of abuse or addiction, or relapse of insomnia after withdrawal. More recently developed treatments, and most notably CBT-I, have shown more sustained positive effects, as indicated by increased sleep efficiency and reduced severity of insomnia complaints [12–14]. Internet-based CBT-I has similar positive effects [15–18]. CBT-I consists of both cognitive (e.g., rumination and worrying) and behavioral (e.g., sleep hygiene, stimulus control, and sleep restriction) components [6]. It aims to change a person’s convictions and beliefs about sleep, and to provide behavioral tools for a better management of sleep [19]. Although CBT-I has proven to be effective in reducing insomnia complaints, it is estimated that only about 60% of the treated people show a clinically significant response among whom only about 40% fully remit [12]. In responders, 50–70% of the complaints may not disappear [20]. Meta-analysis indicates that it is not common for CBT-I to increase sleep efficiency to values well above 85% [17], which is commonly considered as the cutoff for a normal sleep efficiency. Thus, in spite of the undisputed effectiveness of CBT-I, there is ample room for further improvement.

A class of treatments that may help improve effectivity are chronobiological treatments, which aim to support the biological clock of the brain, an essential part of sleep regulation [21]. Circadian rhythms, i.e. the 24-h cycles of physiology and behavior, are entrained by ‘Zeitgebers’, which can be both internal and external stimuli, including light, temperature, and physical activity [22]. CT aims to boost the timed occurrence of these ‘Zeitgebers’. Light therapy and physical activity have shown moderately positive effects on sleep parameters in people with ID [23, 24]. Well-timed body warming, by mild physical activity or taking a hot bath, can support the 24-h rhythm in core body temperature. Body warming can improve sleep quality [25]. CT is of interest as well for the aim of preventing depression in people with insomnia as well, because one of the proposed contributions of insomnia to depression is that poor sleep may lead to circadian dysregulation, which predisposes to depression [3].

The theoretical mechanisms underlying CBT-I and CT can be viewed within the two-process model of sleep regulation proposed by Borbely et al. (1982) [26], which proposes that sleep pressure concerns a homeostatic and a circadian process (commonly denoted, respectively as process S and C). During CBT-I the homeostatic sleep pressure is build up via sleep restriction, while imposed adherence to fixed bedtimes is presumed to support the circadian rhythm [27]. CT aims to further entrain and strengthen the circadian rhythm by increasing its amplitude by means of scheduled light exposure, physical activity and body warming [27]. Indeed, both CBT-I and CTs have proven effective to reduce depressive symptoms. A large RCT in insomnia patients found that internet-based CBT-I ameliorated subclinical depression symptoms [28]. Other studies have shown similar results confirming that CBT-I is effective in reducing both insomnia and depressive symptoms [29, 30] and can even prevent a depressive episode at 1 year follow up [31]. Interestingly, a study comparing CBT for insomnia and CBT for depression in comorbid insomnia and depression patients, found that CBT for insomnia was equally effective in reducing depression severity compared with CBT for depression, while more effective in reducing insomnia complaints [32, 33]. Light therapy, probably the strongest of the different CTs, has also demonstrated to ameliorate depressive symptoms not only in patients suffering from seasonal affective disorder, but also in patients with non-seasonal depression [34].

The recent finding that insomnia comes in different subtypes [8] could propel the development of individualized treatment: what is effective for one subtype may not be effective for another subtype. For the three insomnia subtypes that are most prone to develop depression, it is highly relevant to evaluate whether CBT-I or CT interventions aimed at sleep improvement could also prevent worsening of depressive symptoms or the onset of a major depressive episode.

### Objectives

The current RCT primarily aims to evaluate the efficacy of internet-guided CBT-I, CT, and their combination on the development of depressive symptoms during the year following intervention in people with insomnia of the subtypes that have a high risk of developing depression. The RCT will also evaluate how the treatments affect insomnia symptoms during the year following intervention, and explore how the interventions affect brain structure and function as assessed with Magnetic Resonance Imaging (MRI).

The RCT is embedded in a more extensive study aimed at a better understanding of causes and consequences of insomnia. Extensive data will be assessed to explore multivariate predictors of the development of depression and insomnia, as well as treatment responses, in the year following intervention. Moreover, the same extensive data will be assessed in controls without sleep complaints and people with insomnia of the subtypes that do not have an increased risk of developing depression. This will allow us to explore multivariate differences across controls and insomnia subtypes. The current manuscript focuses on the RCT within this extensive study.

## Methods/design

### Research design

**Fig. 1:**
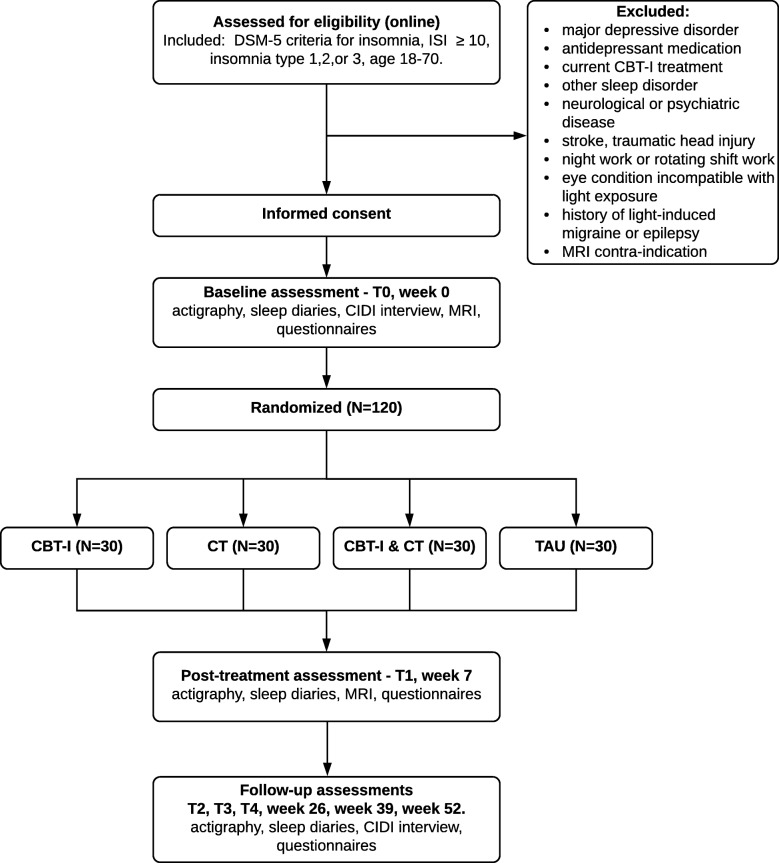
Flowchart diagram showing a summary of the study design. DSM-5: Diagnostic and Statistical Manual of Mental Disorders, 5th edition; ISI: Insomnia Severity Index. CIDI: Composite International Diagnostic Interview; CBT-I: Cognitive Behavioral Therapy for Insomnia; CT: Chronobiological Therapy; TAU: treatment as usual

The study is a parallel-group repeated measures RCT examining the efficacy of CBT-I, CT, and their combination, versus treatment as usual. At baseline (T0), participants of the subtypes with increased risk of developing depression are assessed for baseline values, and randomized to one of the four intervention groups, and will be followed up after completion of the intervention at T1 (week 7), and subsequently at T2 (week 26), T3 (week 39) and T4 (week 52). Figure 1 shows a flow chart of the research design. The study was approved by the Medical Ethics Committee of the VU University Medical Centre (NL63139.029.17). Participants are included only after their written informed consent. The trial is registered with the Netherlands Trial Register (NL7359).

### Participants

The RCT aims to include 120 people with ID of the subtypes with increased risk of developing depression. Inclusion criteria are: 1) meeting the criteria of ID according to the Diagnostic and Statistical Manual of Mental Disorders, 5th edition (DMS-5) [35], and the International Classification of Sleep Disorders, 3rd edition (ICSD-3) [36]; 2) an Insomnia Severity Index score (ISI) ≥ 10 [37]; 3) classification as insomnia type 1, 2, or 3 according to the Insomnia Type Questionnaire [8]; and 4) age between 18 and 70 years. Exclusion criteria are: a) current major depressive disorder according to self-report, a clinical diagnosis, or the Composite International Diagnostic Interview Short Form (CIDI-SF) [38]; b) current treatment with antidepressant medication; c) current CBT-I treatment; d) a diagnosis of or probable severe obstructive sleep apnea syndrome (Apnea Hypopnea Index ≥15 [39], or high risk according to the Berlin questionnaire [40]), moderate to severe restless legs syndrome (International Restless Legs Scale > 15 [41]), or severe periodic limb movement disorder (Periodic Limb Movement Index ≥ 25); e) a self-reported diagnosis of a severe neurological or psychiatric disorder; f) self-reported severe physical or mental impairment due to stroke, or traumatic head injury; g) night work or rotating shift-work; h) a known eye condition incompatible with light exposure; i) a history of light-induced migraine or epilepsy, or severe side effects to bright light in the past; or j) MRI contraindications such as non-MR compatible metal implants, claustrophobia, or pregnancy. The use of sleep medication is allowed and monitored. Participants will be recruited among the participants of the Netherlands Sleep Registry (www.sleepregistry.org), through media advertisements, and through flyers. It will be documented whether participants were recruited from the sleep registry or via media advertisements and flyers.

### Randomization

Participants of the RCT will be randomized to one of the four groups: CBT-I, CT, combination of CBT-I and CT, and treatment as usual (*N* = 30 for each group). Simple randomization will be applied until 2 to 4 participants have been randomized to each of the four groups. In order to ascertain balanced and equally sized groups across the intervention conditions, subsequent participants will be assigned according to covariate adaptive randomization [42]. Matching variables include age, sex, insomnia type, baseline severity of insomnia (ISI [37]) and depressive symptoms (Inventory of Depressive Symptoms – Self Report, IDS-SR [43]), use of sleep medication, suspected mild sleep disordered breathing, suspected mild restless leg syndrome, Body Mass Index (BMI), time of year at inclusion, and group size at inclusion. Since matching includes both continuous and categorical covariates, randomization will be done following the method proposed by Frane [44]. This method evaluates between-group differences for each of the covariates in case a new participant would be assigned to each of the treatment groups by obtaining *P* values from analysis of variance (ANOVA) for the continuous covariates and Chi-square tests for the categorical covariates. For each covariate, this procedure provides four *P* values, one for each possible group assignment. This smallest of all P values identifies the least desirable assignment. Of the four possible group assignments, the most desirable will be the one that maximizes the smallest *P* value across all covariates. This will be the final group assignment for that participant, because it keeps imbalance over groups as small as possible. The randomization is performed using R [45].

### Blinding

Participants of the RCT will be informed of their intervention condition after all baseline assessments at T0 are completed. The research team will be blind to treatment allocation during the baseline assessments at T0. The team member who runs the R script that allocates participants to one of the treatment groups, will not be blinded to the intervention conditions, but is unaware of therapy conditions when communicating with the participants. The CIDI interviewers will be blind to intervention conditions and will be independent from the therapy coaches. During treatment, therapy coaches will not be blind to the intervention conditions, since they have to provide the participants with feedback on the therapy sessions. During data analyses, all data will be coded regarding intervention condition and analysts will not have access to the key document. Only after statistical analyses are completed, conditions may be revealed.

### Assessment points and measures

In participants of the RCT, repeated assessments take place at baseline (T0), during the 6-week treatment period (T_intervention_), and at four follow-up time points (T1, week 7; T2, week 26; T3, week 39; T4, week 52). Table 1 shows the assessments for each time point (according to the Standard Protocol Items: Recommendations for Interventional Trials (SPIRIT) guidelines; the full SPIRIT Checklist is available as Additional file 1). For all participants, the baseline assessments (T0) include an MRI scan, 9 days of actigraphy monitoring and sleep diaries (Consensus Sleep Diary, CSD [46]), and questionnaires assessing demographics, mood (IDS-SR [43]; Hospital Anxiety and Depression scale, HADS [47]; Positive Affect Negative Affect Scale, PANAS [48]), anxiety (Beck Anxiety Inventory, BAI [49]), sleep (ISI [37]; Pittsburgh Sleep Quality Index, PSQI [50]; Duke Structured Interview for Sleep Disorders for DSM-5, DSISD [51]; sleep history; Munich Chrono Type Questionnaire, MCTQ [52]; reduced Morningness-Eveningness Questionnaire, rMEQ [53]), life events (Life Experience Survey, LES-B [54]), childhood trauma (Childhood Trauma Questionnaire, CTQ [55]), physical exercise (Baecke questionnaire [56]), and cost-effectiveness (Trimbos and iMTA questionnaire on Costs associated with Psychiatric Illness, TIC-P [57]). A diagnostic interview (CIDI-SF [38]) is administered to assess a diagnosis of major depressive disorder according to the DSM-IV criteria [58].

**Table 1:** Overview of interventions, assessments, and timepoints

		Study Period					
	Enrolment	Baseline	Intervention	Follow-up assessments			
**Timepoint**	**-t**_1_	**T**_0_	**T**_intervention_	**T**_1_	**T**_2_	**T**_3_	**T**_4_
		*Week 0*	*Week 1–6*	*Week 7*	*Week 26*	*Week 39*	*Week 52*
**Erolment:**							
**Eligibility screen**	X						
**Informed consent**	X						
**Allocation**		X					
**Interventions:**							
***Cognitive Behavioral Therapy for Insomnia (CBT-I)***			X				
***Chronobiological Therapy (CT)***			X				
***Combination of CBT-I & CT***			X				
***Treatment as Usual***			X				
**Assessments:**							
***Demographics***		X					
***CIDI-SF***^1^		X					X
***IDS-SR***^2^		X		X	X	X	X
***HADS***^3^		X		X	X	X	X
***PANAS***^4^		X		X	X	X	X
***Actigraphy***		X		X			X
***CSD***^5^		X	X	X			X
***ISI***^6^	X	X		X	X	X	X
***PSQI***^7^		X					
***DSISD***^8^		X					
***Sleep history***		X					
***MCTQ***^9^		X					
***rMEQ***^10^		X					
***ITQ***^11^	X				X		X
***BAI***^12^		X		X	X	X	X
***LES-B***^13^		X			X	X	X
***CTQ***^14^		X					
***TIC-P***^15^		X					X
***Baecke***		X					
***Compliance***			X				
***Use of interventions***					X	X	X
***Health care use***					X	X	X
***MRI***^16^		X		X			

^1^ Composite International Diagnostic Interview – Short Form, ^2^ Inventory of Depressive Symptoms – Self Report, ^3^ Hospital Anxiety and Depression Scale, ^4^ Positive Affect Negative Affect Scale, ^5^ Consensus Sleep Diary, ^6^ Insomnia Severity Index, ^7^ Pittsburgh Sleep Quality Index, ^8^ Duke Structured Interview for Sleep Disorders, ^9^ Munich Chronotype Questionnaire, ^10^ reduced Morningness-Eveningness Questionnaire, ^11^ Insomnia Type Questionnaire, ^12^ Becks Anxiety Inventory, ^13^ Life Experience Survey, ^14^ Childhood Trauma Questionnaire, ^15^ Trimbos and iMTA questionnaire on Costs associated with Psychiatric Illness, ^16^Magnetic Resonance Imaging

During treatment (T_intervention_), participants randomized to CBT-I (and in combination with CT) fill out the CSD every day during week 1–6 (T_intervention_). Participants randomized to CT (and in combination with CBT-I) fill out a questionnaire about the compliance to the chronobiological interventions every day during week 1–6 (T_intervention_).

The assessments at T1 (week 7) include an MRI scan, 7 days of actigraphy monitoring and sleep diaries (CSD), and questionnaires assessing mood (IDS-SR, HADS, PANAS), anxiety (BAI), and insomnia (ISI). At T2 (week 26) and T3 (week 39) participants fill out questionnaires assessing mood (IDS-SR, HADS, PANAS), anxiety (BAI), insomnia (ISI), insomnia types (ITQ), life events (LES-B), use of therapy interventions and use of health care. After 1 year (T4) participants wear an actigraph for 7 days, fill out sleep diaries for 7 days (CSD), are interviewed to assess a diagnosis of major depressive disorder (CIDI-SF), and fill out questionnaires assessing mood (IDS-SR, HADS, PANAS), anxiety (BAI), insomnia (ISI), insomnia types (ITQ), life-events (LES-B), cost-effectiveness (TIC-P), use of therapy interventions, and use of health care.

### MRI data acquisition

MRI data are acquired using a 3-Tesla MRI scanner with a 32-channel head coil (Philips Achieva, Best, the Netherlands). The following MRI scans are obtained: 1) a T1 weighted image according to the ADNI protocol [59] (repetition time (TR)/ echo time (TE) = 6.5/2.9 ms, voxel size = 1 mm^3^, 211 slices, field of view (FOV) = 256x256x11mm, flip angle = 9°); 2) task-based and 3) resting state functional MRI scans (gradient-echo planar imaging, multi-band factor 4, TR/ TE = 700/30 ms sec, voxel size = 2.7 mm^3^, 44 slices, FOV = 216x216x118mm, flip angle = 55°); 4) Diffusion Weighted Imaging (multi-band factor 2, TR/ TE = 4683/ 95 ms, voxel size = 2 mm^3^, 66 slices, FOV = 224x224x132mm, flip angle = 90°, b = 1000, 29 directions, b = 2000, 59 directions). The task administered during the tasked-based fMRI is from the Human Connectome Project [60] and measures the Blood Oxygen Level Dependent response (BOLD) related to emotional processing. Participants are presented with pictures of emotional faces or neutral shapes. Participants are instructed to indicate which of the two faces or shapes matches the target image. During assessment of resting-state fMRI, participants are looking at a fixation cross for 12 min. Participants are instructed to let their mind wander, think of nothing in particular, and try not to fall asleep. The participant’s eye is video recorded to monitor if participants fall asleep during the scan. The same MRI protocol is used for both the baseline measurement (T0) and the follow-up assessment at week 7 (T1).

### Intervention

Participants will be randomized to one of the following groups: CBT-I, CT, their combination, or treatment as usual.

#### CBT-I

Participants receive guided internet-based, cognitive behavioral therapy (i-Sleep) [15] for 6 weeks. i-Sleep is an internet-delivered CBT-I guided by a personal coach. The i-Sleep treatment module proved to be feasible, acceptable, and effective [15]. i-Sleep consists of 5 weekly sessions to be completed in at most 6 weeks and includes the cognitive and behavioral components that are common for CBT-I. In detail, the five sessions address: 1) Psychoeducation about normal sleep and insomnia, and about sleep hygiene including information about behaviors that are known to stimulate or hamper sleep; 2) stimulus control and sleep restriction: behavioral instructions are given to re-associate the bed/bedroom with sleep and to restrict the time spent in bed to the average amount of night-time sleep; 3) worrying and relaxation: Techniques to reduce worrying and muscle relaxation exercises are proposed; 4) erroneous cognitions about sleep: the basics of cognitive therapy are explained and the most common erroneous ideas about insomnia are addressed; 5) summary and plans for the future. As part of the i-Sleep module, participants complete a sleep diary (CSD) every day.

Each of these sessions contains information, examples, and homework. After finishing each session, the participant receives a personal feedback report by a personal coach, consisting of feedback on participant’s evaluation of previous exercises, their proposed plans, clarifying information and motivating participants to persist in carrying out the requested behavioral changes.

#### CT

Participants receive internet-based CT therapy (i-Cycle) for 6 weeks. i-Cycle is an internet-delivered chronobiological therapy guided by a personal coach. The i-Cycle module is set up in such a way that i-Sleep and i-Cycle can be completed in parallel. Similar to the i-Sleep module, the i-Cycle module consists of 5 weekly sessions that can be completed in at most 6 weeks and includes different components that are commonly considered to support entrainment of the biological clock of the brain. In detail, the five i-Cycle sessions address: 1) Psychoeducation about the circadian rhythm, and about the effect of day and night light exposure on sleep. Participants are taught how to bring structure into their sleep-wake cycle and receive instructions for daily use of the Philips EnergyUp HF3430/01 lamp, shortly after awakening for 30 min at a distance of 40 cm (from eye to lamp); 2) Physical activity: in addition to using the light, participants are informed about the effect of physical activity on sleep and are encouraged to exercise more frequent than usual and/or at a fixed time of day; 3) Physical activity: participants continue the use of scheduled light and exercise while trying to increase the intensity of their physical activity; 4) Temperature: participants receive information indicating that sleep propensity can be enhanced by warming the skin and are instructed to take a warm bath (or a hot shower if no bath is available), for approximately 3 times a week, in the interval 3 to 2 h before bedtime, for 30 min, at a temperature ~ 37–39 °C); 5) Summary and plans for the future. As part of the i-Cycle module a daily activity diary is administered to assess compliance to the interventions.

Each of these sessions contains information, examples, and homework. After finishing each session, the participant receives a personal feedback report by a personal coach, consisting of feedback on participant’s evaluation of previous exercises, their proposed plans, clarifying information and motivating participants to persist in carrying out the requested behavioral changes.

#### Combination of CBT-I and CT

Participants receive both CBT-I and CT as described above.

#### Treatment as usual

Participants do not receive i-Sleep or i-Cycle, but are allowed to seek help from their general practitioner or other specialist (health care use is monitored).

### Outcome measures

The primary outcome is the severity of depressive symptoms during the year following the intervention relative to baseline. Depressive symptoms are repeatedly assessed with the IDS-SR. Mixed effects regression analysis will estimate the integrated treatment effect on all four follow-up IDS-SR assessments from T1 (7 weeks, directly after the six-week intervention) to T4 (52 weeks), relative to T0 (baseline).

### Secondary measures

A secondary outcome is a diagnosis of major depressive disorder based on the CIDI-SF at T4 (52 weeks) relative to T0 (baseline). Other secondary outcome measures are the severity of insomnia and the cost-effectiveness of each treatment calculated from health care and work absenteeism. Severity of insomnia will be measured with the ISI, CSD and actigraphy recordings. Cost-effectiveness will be assessed with the TIC-P questionnaire. Another secondary outcome measure is the effect of the intervention (CBT-I and/or CT) on brain structure and function (assessed with MRI) at T1 relative to T0.

### Sample size calculation

Sample size calculations for mixed effect regression models require a number of estimates, or assumptions [61]. We estimate an average dropout of 3% with every next assessment (all-completers at T0, 3% drop-out at T1 to 12% drop-out at T4). We estimate the (within-subject) intraclass correlation coefficient (ICC) of the pre-assessment and four follow-up assessments to be approximately 0.50. A somewhat higher ICC will slightly inflate the minimal detectable effects, a somewhat lower ICC will slightly lower the minimal detectable effects. In the two by two factorial intervention design, the ratio of participants who will receive CBT-I versus those who will not is 1. This is also the case for those who will receive CT versus those who will not. For the interaction effect of CBT-I x CT, the ratio of participants who will receive both interventions versus those who will not is 1/3. We follow Cohen’s suggestion that an effect size below *d* = 0.2 (small) is not likely to be clinically significant, that *d* = 0.5 represents a medium effect size, and that *d* = 0.8 represents a large effect size [62]. A feasible total number of completers (i.e. complete data on the ambulatory recordings, EEG sleep recordings, MRI and questionnaires) at baseline is *N* = 120, *N* = 30 for each of the four groups, of whom we assume that *N* = 26 per group will complete all follow-up assessments. At a significance threshold of *α*  = 0.05, this sample size provides a power 1-*β*  = 0.80, the minimal detectable interaction effect is *d* = 0.5, while the minimal detectable main effects of either CBT-I or CT are *d* = 0.44 (medium). A previous similar study reported a more than medium effect of CBT-I on depressive symptoms at T1 (*d* = 0.74) up to a large effect at T4 (*d* = 1.12) [63].

### Statistical analyses

Statistical analyses aim at determining the relative efficacy of internet-guided CBT-I, CT treatments and their combination, for the prevention of worsening of depressive symptoms in people with the type of ID that heralds increased risk of developing depression. The long-term effects of interventions and their interactions on depressive symptoms and on the probability to develop a diagnosis of depression will be estimated with mixed effect linear and logistic regression models using R [45].

In order to allow for an intention-to-treat analysis including all available data while accounting for a possibly variable number of missing data, hierarchical mixed effect linear and logistic regression models will be employed. Regressors are a random intercept, time, dummy-coded assignment to CBT (yes = 1, no = 0) and CT (yes = 1, no = 0) and a dummy coded variable indicating follow-up (T1-T4 = 1) versus baseline (T0 = 0). The interaction effect estimates of interest are CBT by follow-up, CT by follow-up and CBT-I by CT by follow-up [64]. The time variable estimates linear changes over time. Ancillary analyses will evaluate treatment by time effects (i.e., treatment effects changing linearly over time). Linear mixed effect models will also be used evaluate secondary outcomes (insomnia severity, cost-effectiveness, brain function and structure). A logistic mixed effect model will be used to evaluate the secondary outcome of a diagnosis of depression at T4. We will explore the effect of various variables, including the number of previous depressive episodes, time since last depressive episode, and expectation of intervention effectiveness.

### Ancillary assessments

This trial is embedded in a larger study, where we aim to explore the multivariate predictors of the development of depression in people with ID. Apart from the 120 participants with ID with a subtype prone to develop a depression, we include 30 people with ID of one of the two subtypes that is less prone to develop a depression [8], and 30 age and sex matched controls. Ancillary assessments at baseline (T0) include 9 days of experience sampling and ambulatory recordings (actigraphy, light exposure, heart rate), and one night of High-Density (256 channel) Electroencephalography (HD-EEG) sleep recordings.

### Limitations

Our study is limited by the fact that internet-based therapy might not be suitable for everyone (e.g. elderly people). Although internet-based therapies have been reported to establish near equal effectiveness as face-to-face therapy [18], some individuals might prefer face-to-face therapy over internet-based therapy. It would also be interesting to investigate if treatment response differs between the three insomnia subtypes included in the RCT, however our sample size will not be sufficient if subtype differences are too small.

## Discussion

People with ID are at risk of worsening of depressive symptoms and developing a major depressive episode. Pre-selection of three high-risk insomnia subtypes allows for a sensitive assessment of the possibility to prevent the development and worsening of depressive symptoms through interventions targeting insomnia. This trial will compare the efficacy of CBT-I, CT and their combination, to prevent worsening of depressive symptoms by treating insomnia symptoms.

### Trial status

Data collection started on December 6, 2018 and and will continue until October 1, 2020.

## Supplementary information

**Additional file 1.** SPIRIT Checklist.

## Data Availability

The data collected in this study will be available from the corresponding author upon reasonable request and after publication of findings.

## Abbreviations

ANOVA: Analysis of variance
BAI: Beck Anxiety Inventory
BOLD: Blood Oxygen Level Dependent response
CBT-I: Cognitive Behavioral Therapy for Insomnia
CIDI-SF: Composite International Diagnostic Interview Short Form
CSD: Consensus Sleep Diary
CT: Chronobiological Therapy
CTQ: Childhood Trauma Questionnaire
DSISD: Duke Structured Interview for Sleep Disorders
DSM: Diagnostic and Statistical Manual of Mental Disorders
FOV: Field of View
HADS: Hospital Anxiety and Depression scale
HD-EEG: High-Density Electroencephalography
ICC: Intraclass correlation
ICSD: International Classification of Sleep Disorders
IDS-SR: Inventory of Depressive Symptoms – Self Report
ISI: Insomnia Severity Index
LES-B: Life Experience Survey
MCTQ: Munich Chronotype Questionnaire
MRI: Magnetic Resonance Imaging
PANAS: Positive Affect Negative Affect Scale
PSQI: Pittsburgh Sleep Quality Index
RCT: Randomized Controlled Trial
rMEQ: Reduced Morningness-Eveningness Questionnaire
TAU: Treatment as usual
TE: Echo time
TIC-p: Trimbos and iMTA questionnaire on Costs associated with Psychiatric Illness
TR: Repetition time
